# Preclinical carotid atherosclerosis in patients with latent autoimmune diabetes in adults (LADA), type 2 diabetes and classical type 1 diabetes

**DOI:** 10.1186/s12933-017-0576-9

**Published:** 2017-07-28

**Authors:** Marta Hernández, Carolina López, Jordi Real, Joan Valls, Emilio Ortega-Martinez de Victoria, Federico Vázquez, Esther Rubinat, Minerva Granado-Casas, Nuria Alonso, Teresa Molí, Angels Betriu, Albert Lecube, Elvira Fernández, Richard David Leslie, Dídac Mauricio

**Affiliations:** 1 Department of Endocrinology and Nutrition, University Hospital Arnau de Vilanova, Lleida, Spain; 2 Nursing School, Universitat de Lleida, Lleida, Spain; 3 Institut de Recerca Biomèdica de Lleida, Lleida, Spain; 4 Unitat de Suport a la Recerca Lleida, Institut Universitari d’Investigació en Atenció Primària Jordi Gol (IDIAP Jordi Gol), Barcelona, Spain; 5 Epidemiologia i Salut Pública, Universitat International de Catalunya, Sant Cugat del Vallés, Spain; 6 Department of Basic Medical Sciences, Universitat de Lleida, Lleida, Spain; 7 Department of Endocrinology and Nutrition, CIBEROBN-Spanish Biomedical Research Centre in Physiopathology of Obesity, Hospital Clínic, Barcelona, Spain; 8 Department of Endocrinology and Nutrition, CIBER of Diabetes and Associated Metabolic Diseases, Health Sciences Research Institute & University Hospital Germans Trias i Pujol, Carretera Canyet S/N, Badalona, 08916 Spain; 9 UDETMA, Department of Nephrology, University Hospital Arnau de Vilanova, Lleida, Spain; 10 The Blizard Institute, Barts and the London School of Medicine and Dentistry, London, UK

**Keywords:** Carotid plaque, Atherosclerosis, Late onset autoimmune diabetes, LADA, Type 1 diabetes, Type 2 diabetes

## Abstract

**Background:**

LADA is probably the most prevalent form of autoimmune diabetes. Nevertheless, there are few data about cardiovascular disease in this group of patients. The aim of this study was to investigate the frequency of carotid atherosclerotic plaques in patients with LADA as compared with patients with classic type 1 diabetes and type 2 diabetes.

**Methods:**

Patients with LADA were matched for age and gender in different proportions to patients with type 2 diabetes, and classic type 1 diabetes. None of the patients had clinical cardiovascular disease. All subjects underwent B-mode carotid ultrasound to detect atheroma plaques. Demographics were obtained from all subjects.

**Results:**

We included 71 patients with LADA, 191 patients with type 2 diabetes and 116 patients with type 1 diabetes. Carotid atherosclerosis was more frequent in patients with LADA compared with type 2 diabetes (73.2% vs. 56.9%, *P* = 0.0018) and classic type 1 diabetes (57.1%, *P* = 0.026); these changes occurred despite healthier macrovascular risk profiles in the former. Age (*P* < 0.001), smoking (*P* = 0.003) and hypertension (*P* = 0.019) were independently associated with carotid atherosclerosis. Multiple plaques were also more frequent in patients with LADA as compared with classic type 1 diabetes and type 2 diabetes (45.1% and 33.6% vs. 27.2%, respectively, *P* = 0.022). The frequency of carotid plaques increased with increasing diabetes duration in LADA patients compared with type 2 diabetes (85.7% vs. 58.8%, inverse OR 5.72 [1.5–21.8]; *P* = 0.009).

**Conclusions:**

LADA patients do not present with less carotid atherosclerosis than patients with type 1 and type 2 diabetes. Their macrovascular risk occurs despite a healthier macrovascular risk profile than those patients with type 2 diabetes.

## Background

Macrovascular disease is the leading cause of morbidity and mortality in patients with both type 1 diabetes and type 2 diabetes [1]. Latent autoimmune diabetes of the adults (LADA), that is patients with adult-onset autoimmune diabetes who do not initially require insulin, represent 4–14% of subjects previously diagnosed with type 2 diabetes [2]. LADA can be estimated to have a higher prevalence than classic type 1 diabetes in both children and adults. Notwithstanding its clinical relevance, data about cardiovascular events and mortality in patients with LADA are limited. It could be anticipated that patients with LADA would be less likely to develop macrovascular disease than patients with type 2 diabetes, as they have a reduced risk of metabolic syndrome [3–5]. However, most studies to date, despite their limited size, population heterogeneity and variable disease duration, have failed to show such a reduced risk, and indeed many find an increased risk [6–9].

Preclinical atherosclerosis can be detected and quantified non-invasively by carotid ultrasound, which is a strong predictor of cardiovascular events [10–16]. Although there is still controversy in this field, established carotid atherosclerosis (carotid plaques), detected by ultrasound imaging, improves risk stratification when added to traditional risk factors. Indeed, individuals with carotid plaques are considered a very-high risk category according to the most recent guidelines, and carotid artery plaque assessment using ultrasonography may be considered to be a valid risk modifier in cardiovascular prediction [17–19]. Patients with diabetes mellitus are at high risk of cardiovascular events; therefore, the added potential of carotid plaque detection in risk assessment would be different from those cases without diabetes [20–22]. However, primary prevention targets are often not reached in patients with diabetes, and carotid plaque detection could focus more intensive preventive strategies in these cases, especially in those patients without known organ damage or important associated cardiovascular risk factors [18, 19, 23].

The current study was initially designed to assess the difference in preclinical carotid plaque burden in patients with LADA, as compared to type 2 diabetes. After the results of the initial study, we extended the comparison to age and gender matched patients with classic onset type 1 diabetes.

## Methods

This was a cross-sectional study to evaluate carotid atherosclerotic disease in patients with LADA in comparison with patients with type 2 diabetes and classic onset type 1 diabetes.

### Study subjects

All adult patients attending local diabetes outpatient clinics in the province of Lleida, Spain, who were screened for diabetes-associated autoantibodies and defined as LADA if they had diabetes diagnosed over 30 years of age, with positive glutamic acid decarboxylase (GAD) antibodies and without need of insulin treatment in the first 6 months after diagnosis [24]. From this cohort of 80 LADA patients, we invited all the patients that fulfilled the inclusion criteria (n = 71, acceptance rate: 100%): absence of clinical cardiovascular disease and without established diabetic nephropathy [urine albumin-to-creatinine ratio (UACR) < 300 mg/g and estimated (MDRD4) glomerular filtration rate (eGFR) > 60 ml/min/1.73 m^2^].

Age- and sex-matched subjects with type 2 diabetes were randomly selected to a 3:1 proportion and for classic onset type 1 diabetes on a 2:1 proportion from the same local cohort using the same cardiovascular and renal inclusion criteria. All patients selected with type 2 diabetes were negative for GAD antibodies.

The absence of clinical macrovascular disease in all subjects was confirmed by clinical assessment and review of patients’ medical records to verify absence of heart failure, cerebrovascular disease, coronary heart disease, or peripheral arterial disease (all of them clinically assessed, including any form of diabetic foot disease).

All the participants signed an informed consent form and the Ethics Committee of both participant centers approved the study.

### Clinical assessment

For each subject, age, sex, weight, height, body mass index and waist circumference were measured by standardized methods. Blood pressure (mean of 2 measurements 5 min apart) was measured using a blood pressure monitor (HEM-7001E, Omron, Spain) after 10 min in the seated position. Patients were specifically interviewed about the treatment of diabetes and smoking habit. Former smokers were those who had quit at least for 1 year before enrollment. A patient was arbitrarily considered to have previous hypertension or dyslipidemia if she/he was taking medication for the given condition.

Fasting blood and urine samples were collected and analyzed locally, using standardized assays to measure glucose, glycosylated haemoglobin, the lipid profile [including high-density lipoprotein (HDL) cholesterol, low-density lipoprotein (LDL) cholesterol and triglycerides] creatinine and the albumin-to-creatinine ratio. Glomerular filtration rate was estimated by the modification of diet in renal disease formula (MDRD4). GAD antibodies were measured with a commercially available ELISA kit (DRG Diagnostic, Marburg, Germany), as previously described [25]. Optimal cut-off value for positivity was set at 5 U/ml. The assay showed good performance when tested in the Diabetes Antibody Standardization Program (DASP) Workshops. In DASP 2007, sensitivities and specificities for GAD antibodies were 94 and 97% and in DASP 2009 were 82 and 95%, respectively.

### Carotid ultrasound imaging

All the study participants underwent the same carotid ultrasound protocol. Carotid ultrasound imaging was performed using a high resolution B-mode ultrasound (Sequoia 512, Siemens, North Rhine-Westphalia, Germany) equipped with a 15-Mhz linear array probe. A standardized imaging protocol was performed to evaluate intima-media-adventitia thickness (IMT) as has been described before [26]. The analysis of the presence of atheromatous plaques was performed indistinctly by two readers in a non-blinded fashion. Plaques were identified using B-mode and color Doppler examinations in both the longitudinal and transverse planes to consider circumferential asymmetry. Plaques were defined according to the Mannheim consensus [27]. We examined bilateral carotid arteries (common, bifurcation and internal) to evaluate the presence of plaques. Subclinical carotid atherosclerosis was defined as the presence of at least one plaque in any of the carotid territories explored. The presence of multiple plaques, defined by the presence of plaques in more than one of the explored territories, was considered to reflect more severe atherosclerotic disease [28, 29].

### Sample size

For the comparison of LADA and classic type 1 diabetes with type 2 diabetes, we hypothesized a carotid plaque frequency of 50% for the former two and 70%, for the latter; the latter was based on the known frequency in our local cohort, and the former on a presumed reduced frequency. We calculated that with LADA (n = 62) and type 2 diabetes (n = 186) we would have 80% power, at *P* < 0.05 to detect differences. Using the 2:1 proportion for classic type 1 diabetes we calculated a minimum sample size of 104 subjects (80% power, *P* < 0.05).

### Statistical analysis

A comparison of the characteristics between groups was performed expressing the quantitative variables as mean and standard deviation, and qualitative variables as frequencies and percentages. The statistical significance of differences between groups was assessed using the Chi square test for comparison of proportions, and analysis of variance (ANOVA) for comparison of means. Multiple pairwise comparisons were performed using Bonferroni correction. To assess whether the frequency of plaque and/or multiple plaque was associated with the type of diabetes the crude Odds Ratio was calculated and subsequently adjusted with the respective confidence intervals at 95%. The adjustment variables, besides the type of diabetes, were duration of diabetes, age, sex, smoking, hypertension, dyslipidemia and retinopathy at the time of inclusion. Age and duration of diabetes were categorized according to tertiles, and adjusted models were introduced by functions smoothing splines, with a focus on automatic smoothing parameter selection. The adjusted ORs were estimated using generalized additive models with logit link with package “gam” [30, 31] from R 3.2.1 statistical software [32]. The goodness of fit of the models was assessed with the Hosmer–Lemeshow test. *P* values less than 0.05 were considered statistically significant.

## Results

We recruited 71 patients with LADA, 191 patients with type 2 diabetes and 116 patients with type 1 diabetes. The clinical characteristics of the study groups are shown in Table 1. Patients with LADA, classic type 1, and type 2 diabetes were similar in age, gender distribution, current smoking status, use of antihypertensive drugs, and HbA1c concentrations. As patients were matched by age, the diabetes duration was longer in patients with type 1 diabetes than in patients with LADA, but also, in the latter duration was longer than in type 2 diabetes (23.7 ± 12.4, 13.2 ± 9.7 and 8.7 ± 7.9 years, respectively, *P* < 0.001). While the prevalence of diabetic retinopathy, as expected given the longer disease duration, was higher in type 1 diabetes than in LADA and type 2 diabetes, the frequency of microalbuminuria was similar. About half the patients with LADA were treated with metformin, and the majority were receiving insulin. Patients with type 2 diabetes, as expected, exhibited a more adverse lipid profile and anthropometric measures than the other two cohorts, though fewer were on statin treatment.

**Table 1:** Characteristics of study subjects Table 1

Variables	Type 1 diabetes (n = 116)	LADA (n = 71)	Type 2 diabetes (n = 191)	*P* value
Age (years)	56.5 ± 10.8	58.3 ± 11.6	58.3 ± 10.5	0.321
Male n (%)	52 (44.8)	37 (52.1)	105 (55)	0.224
BMI (kg/m^2^)	26.5 ± 3.9	27.4 ± 5	31.3 ± 5.3	<0.001^a^
Waist (cm)	91.6 ± 13.3	96.3 ± 14	104.8 ± 11.7	<0.001
Diabetes duration (years)	23.7 ± 12.4	13.2 ± 9.7	8.7 ± 7.9	<0.001
Smoking (current/past/never) n (%)	21 (18.1)/33 (28.4)/62 (53.4)	17 (23.9)/22 (31)/32 (45.4)	41 (21.5)/66 (34.6)/83 (43.5)	0.647
Anti-hypertensive treatment n (%)	54 (46.6)	37 (52.1)	98 (51.3)	0.667
Statin treatment n (%)	73 (62.9)	42 (59.2)	71 (37. 2)	<0.001^a^
Antiplatelet agents n (%)	50 (43.1)	40 (56.3)	59 (30.9)	0.002^b^
Metformin treatment n (%)	0	31 (43.7)	138 (72.3)	<0.001
Insulin treatment n (%)	100	63 (88.7)	37 (19.4)	<0.001
Fasting glycemia (mg/dl)	170.9 ± 79.1	143.8 ± 53.4	151.7 ± 52.6	0.006^c^
HbA1c (%)	7.7 ± 1	7.7 ± 1.1	7.4 ± 1.3	0.07
HbA1c (mmol/mol)	60.6 ± 12.5	61 ± 11.4	57.3 ± 18.6	0.07
Total cholesterol (mg/dl)	184.9 ± 29.8	178.9 ± 37.8	185.6 ± 35.3	0.357
LDL-cholesterol (mg/dl)	102.6 ± 25.4	102 ± 24.8	110.6 ± 29.4	0.015^a^
HDL-cholesterol (mg/dl)	65.9 ± 14.6	61.5 ± 18.2	48.4 ± 12.1	<0.001^a^
Triglycerides (mg/dl)	79.9 ± 36.4	100.5 ± 72.9	141.3 ± 78.7	<0.001^a^
Diabetic retinopathy n (%)	52 (44.8)	13 (18.3)	36 (18.8)	<0.001^c^
Microalbuminuria > 30 mg/g creatinine	10 (8.6)	7 (9.9)	12 (6.3)	0.563
Urinary albumin:creatinine ratio (mg/g)	12.2 ± 29.8	20.1 ± 69.6	14.5 ± 32.9	0.451
Mean IMT	0.750 ± 0.109	0.775 ± 0.147	0.793 ± 0.135	0.047^c^

Values are expressed as the mean ± SD or percentages

*LADA* latent autoimmune diabetes in adults, *BMI* body mass index, *HDL* high-density lipoprotein, *LDL* low-density lipoprotein, *HbA1c* glycosylated haemoglobin

^a^ Type 2 diabetes different from LADA and type 1 diabetes

^b^ Type 2 diabetes different from LADA

^c^ Type 1 diabetes different from LADA and type 2 diabetes

**Fig. 1:**
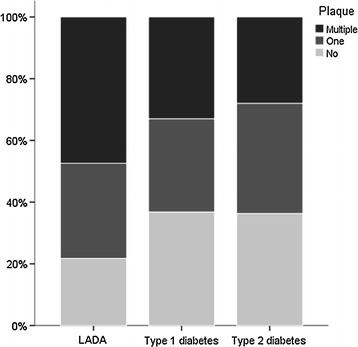
Carotid atherosclerosis in patients with LADA, type 1 and type 2 diabetes. The percentage of patients with carotid plaques was significantly higher in the LADA group (73.2%) than in the group of patients with type 1 diabetes (57.1%, *P* = 0.026) and type 2 diabetes (56.9%, *P* = 0.018). The difference was mainly due to the percentage of patients with multiple plaques, which was higher in LADA (45.1%), than in type 1 diabetes (33.6%), *P* = 0.077 and type 2 diabetes (27.2%), *P* = 0.019. *LADA* latent autoimmune diabetes in adults

For carotid ultrasound findings: mean carotid IMT was lower in patients with type 1 diabetes compared to both LADA and type 2 diabetes (Table 1). As shown in Fig. 1 subclinical carotid atherosclerosis (presence of atherosclerotic plaques) was more frequent in patients with LADA than in patients with type 1 diabetes and type 2 diabetes [73.2% vs. 57.1% (*P* = 0.026) and 56.9% (*P* = 0.018), respectively]. Multiple plaques were also more frequent in patients with LADA than in type 1 diabetes and type 2 diabetes [45.1% vs. 33.6% (*P* = 0.077), and 27.2% (*P* = 0.019), respectively] (Fig. 1).

Carotid plaques were also related to older age, longer diabetes duration, hypertension, retinopathy and dyslipidemia (Table 2).

**Table 2:** Univariate analysis for the presence of carotid atherosclerotic plaques

	Odds ratio (95% confidence interval)	*P* value
Diabetes		
LADA	1	
Type 1 diabetes	0.482 (0.254–0.916)	0.026
Type 2 diabetes	0.486 (0.267–0.884)	0.018
Diabetic retinopathy	1.954 (1.195–3.196)	0.008
Male sex	1.275 (0.844–1.926)	0.248
Dyslipidemia	1.808 (1.192–2.747)	0.005
Hypertension	2.639 (1.721–4.032)	<0.001
Smoker/former smoker	1.464 (0.868–2.469)	0.152
Diabetes duration (tertiles)		
≤5 years	1	
6–11 years	1.360 (0.779–2.375)	0.279
≥12 years	1.875 (1.140–3.084)	0.013
Age (tertiles)		
≤52 years	1	
53–63 years	2.267 (1.371–3.748)	<0.001
≥64 years	3.707 (2.177–6.313)	<0.001

*LADA* latent autoimmune diabetes in adults

A multivariate logistic regression model including age, diabetes duration, gender, type of diabetes, retinopathy, hypertension, smoking and dyslipidemia (Table 3) revealed that age, but not gender, type of diabetes, and the presence of hypertension and smoking habit were associated with carotid atherosclerosis. The adjusted inverse OR for the presence of carotid plaques in patients with LADA as compared with those with type 1 and type 2 diabetes was 2.44 [(1.65–3.6); *P* = 0.0225] and 2.06 [(1.47–2.9); *P* = 0.0334], respectively.

**Table 3:** Adjusted Odds ratio for the presence of carotid atherosclerotic plaques

	Adjusted odds ratio (95% confidence interval)	*P* value
Diabetes		
LADA	1	
Type 1 diabetes	0.410 (0.278–0.606)	0.0225
Type 2 diabetes	0.485 (0.345–0.682)	0.0334
Diabetic retinopathy	1.258 (0.910–1.741)	0.4790
Female sex	0.764 (0.603–0.967)	0.2530
Dyslipidemia	1.252 (0.980–1.599)	0.3587
Hypertension	1.773 (1.389–2.264)	0.0189
Smoker/former smoker	2.471 (1.824–3.349)	0.0029

Odds ratio was adjusted by age and diabetes duration using a generalized additive model

*LADA* latent autoimmune diabetes in adults

To explore the impact of disease duration on carotid plaque frequency in patients with LADA, we limited the analysis to patients with LADA and type 2 diabetes, as few patients with type 1 diabetes had a short diabetes duration. We divided patients with LADA and type 2 diabetes into age-adjusted tertiles of disease duration: ≤5, 6–11, ≥12 years. Table 4 shows the results of this analysis; within 5 years of diabetes duration the prevalence of carotid plaques was lower, though not significantly so, in patients with LADA than in those with type 2 diabetes [26.7% vs. 54.8%, inverse OR 0.38 (0.11–1.39); *P* = 0.137]. However, with increasing diabetes duration the frequency of carotid atherosclerotic plaques increased in patients with LADA, but not in patients with type 2 diabetes [81.8% vs. 50%, inverse OR 5.72 (1.5–21.8); *P* = 0.009].

**Table 4:** Age-adjusted frequency of carotid atherosclerotic plaques in patients with LADA and type 2 diabetes, divided by tertiles of disease duration

Diabetes duration	N	Carotid plaque (%)	Inverse OR (95% confidence interval)	*P* value
≤5 years				
Type 2 diabetes	84	54.8		0.137
LADA	15	26.7	0.38 (0.11–1.39)	
6–11 years				
Type 2 diabetes	60	50		0.009
LADA	22	81.8	5.72 (1.5–21.8)	
≥12 years				
Type 2 diabetes	47	70.2		0.038
LADA	34	88.2	4.19 (1.06–16.6)	

*LADA* latent autoimmune diabetes in adults

## Discussion

In this study, we unexpectedly found an increased frequency of subclinical carotid atherosclerosis in patients with LADA, as compared to subjects with classic type 1 diabetes and type 2 diabetes. That excess frequency of carotid disease was also evident when assessing multiple plaques in LADA compared with the other cohorts. We did not find the same results for cIMT as for carotid plaque, potentially due to the high variability and low intra-individual reproducibility of the former measurement [33]. Moreover, the pathophysiology underlying the development of carotid plaque and cIMT is distinct and the evidence regarding the value of cIMT in cardiovascular risk prediction is contradictory and of debatable value [34–36]. Most recent clinical guidelines on cardiovascular risk do not recommend carotid IMT for individual risk prediction [18, 37]. In contrast, the presence of carotid plaques has been shown to be a good predictor of future cardiac events [15, 18].

The high frequency of carotid plaques, despite the increased use of statins in LADA cases, remained after adjusting for the main cardiovascular risk factors, including diabetes duration. The current cross-sectional study does not allow for the identification of potential causes of our unexpected finding of higher atherosclerotic burden in LADA, even though patients with type 2 diabetes tend to exhibit, as in other studies, a worse cardiovascular risk profile including higher blood pressure, more obesity and an adverse lipid profile. In both the Freemantle Study [38] and the Collaborative Atorvastatin Study [7], patients designated LADA had comparable cardiovascular disease as the patients with type 2 diabetes; while in the Botnia study [6] after 13 years of diabetes 56% of LADA patients had ischemic heart disease and 5% had had cerebrovascular events. To date, only one other study has assessed the frequency of carotid atherosclerosis in patients with LADA and type 2 diabetes [8], and none have assessed that frequency in the three cohorts we assessed. That Chinese study was retrospective and not designed to answer the question we posed here, but they did find a comparable frequency of carotid atherosclerosis compared with type 2 diabetes patients. Taken together, these studies indicate that patients with adult-onset diabetes, irrespective of the cause of the diabetes, are at risk of carotid and coronary atherosclerosis. It is difficult to understand why this should be, given that autoimmune diabetes cases are less likely to have the metabolic syndrome and related features [7]. Two possibilities suggest themselves. First, the time of onset of autoimmune diabetes antedates the disease by months, even years, and it remains possible that LADA patients have had low-grade disease many years before clinical presentation, as can be the case with type 2 diabetes. However, the duration of LADA pre-disease is unknown both in this study and in general, while the risk of atherosclerosis in these cases was increased with increasing diabetes duration post-diagnosis. Second, patients with LADA are not well managed and are consistently shown to have higher HbA1c levels than comparable cohorts of patients with type 2 diabetes [4, 39–41]. Higher HbA1c has been found to be associated with higher coronary and carotid atherosclerotic burden in non-diabetic patients [42]. Additionally, deranged glycemic control has been independently related to coronary heart disease in both LADA patients [6], and diabetes patients in general [40, 43]. Higher HbA1c concentrations have also been related to cardiovascular disease in the non-diabetic population in prospective studies, including the Norfolk cohort of European Prospective Investigation into Cancer and Nutrition study [44] and The Copenhagen City Heart Study [45].

In subjects with diabetes, other potential different pathophysiological mechanisms may be involved in the atherosclerotic process. First, advanced glycation end products (AGEs) which are increased in diabetes are pro-oxidants that induce oxidative stress and are thought to play a major role in atherosclerosis [46, 47]. Although diet is the principal source of AGEs, their concentrations rise with increasing blood glucose. An increased level of advanced AGEs has been associated with cardiovascular disease in type 1 diabetes [48] and type 2 diabetes [49]; no such data is available in LADA. Secondly, patients with systemic autoimmune diseases, such as rheumatoid arthritis or systemic lupus erythematosus, have an increased risk of atherosclerosis, with both cellular and humoral components of the immune system involved in its pathogenesis and some shared genetic risk [50], although the precise mechanisms underlying the high cardiovascular risk in these patients is unclear [51]. It is well known that chronic inflammation plays an important role in atherogenesis [52, 53], and inflammatory markers associated with macrovascular disease are elevated both in patients with type 1 diabetes and type 2 diabetes [54, 55]. Moreover, poor glycemic control and hypoglycaemic episodes are related to pro-inflammatory status [56, 57], and may be additional contributing factors in atherosclerosis in patients with autoimmune diabetes [58]. Unfortunately, in the current study we did not determine any markers of chronic inflammation in the LADA patients, though we have previously demonstrated such changes in LADA patients [59].

Nevertheless, this study has some weaknesses. First of all, the size of the populations studied is relatively small, and some important information is missing, notably the familial history of premature cardiovascular disease. While we found no differences in HbA1c between the three diabetes groups, this is a cross-sectional study, and the relationship between glycemic control and atherosclerosis can only be answered with a prospective design. The situation is made more complex because most of the patients with LADA in this study had already been referred to specialized care, usually because of poor glycemic control. The specialized diabetes care of all the patients with type 1 diabetes and most of the patients with LADA may explain the increased use of statins and antiplatelet agents in these groups compared to that of patients with type 2 diabetes, since the latter are usually under general practitioner’s care. Since LADA patients were recruited from a specialty-based cohort our results may show a bias; however, this group of LADA subjects is likely representative of LADA patients in general in Spain.

Nevertheless, despite its limitations, our study has the strength of comparing for the first time these three groups of diabetes, which come from a homogeneous population. While we use the term LADA throughout the text for clarity, we would prefer that LADA is called adult-onset autoimmune diabetes since there is no firm evidence that LADA can be categorically distinguished from type 1 (autoimmune) diabetes.

## Conclusions

Our results indicate that the frequency of preclinical carotid atherosclerosis in patients with adult-onset initially non-insulin requiring autoimmune diabetes, also called LADA, is comparable, even greater, than in adults of similar age with classic type 1 diabetes and type 2 diabetes. Our data should be confirmed in both cross-sectional and prospective studies in other populations. However, the results draw attention to the importance of macrovascular disease in patients with diabetes, irrespective of the cause of the disease, and emphasize the potential value of rigorous treatment of macrovascular risk factors even in patients with autoimmune diabetes.

## Abbreviations

LADA: latent autoimmune diabetes in adults
IMT: intima-media-adventitia thickness
